# Ιmpact of sunitinib-induced hypothyroidism on survival of patients with metastatic renal cancer

**DOI:** 10.1186/s12885-019-5610-8

**Published:** 2019-04-30

**Authors:** Theofanis Vasileiadis, Michail Chrisofos, Michail Safioleas, Konstantinos Kontzoglou, Konstantinos Papazisis, Athina Sdrolia

**Affiliations:** 1 Theagenion Cancer Hospital, Al. Symeonidi 2, 54007 Thessaloniki, Greece; 2 Urology Department, Αttikon Hospital, Rimini 1, Chaidari, 124 62 Athens, Greece; 3 2nd Department of Propedeutic Surgery, Laiko Hospital, Agiou Thoma 17, 115 27 Athens, Greece; 4 School of Medicine, National Kapodistrian University of Athens, 157 72 Athens, Greece; 5 Present Address: Endocrinology Department, Hull Royal Infirmary, Anlaby Road, Hull, HU3 2JZ UK; 6 Present Address: Oncology Department, Geniki Kliniki, M. Kallas 11 Gravias 2, 546 45 Thessaloniki, Greece; 7 Radiation Physics Department, Queen’s Centre for Oncology and Haematology, Castle Hill Hospital, Castle Rd, Cottingham, HU16 5JQ UK

**Keywords:** Sunitinib, Hypothyroidism, Metastatic renal cell cancer, Clear cell carcinoma, Thyroid-stimulating hormone, TSH

## Abstract

**Background:**

Sunitinib plays an important role in managing the metastatic renal cell cancer (mRCC). Sunitinib-induced hypothyroidism is a common side-effect of the drug. There have been attempts to link hypothyroidism with a better clinical outcome in sunitinib-treated (mRCC) patients. Our aim was to relate the impact of hypothyroidism to the survival of these patients.

**Methods:**

We have evaluated 70 patients with mRCC that received sunitinib as a first line treatment. Thyroid-stimulating hormone (TSH) was measured at baseline, after 15 days of treatment (day-15) and at the end of the second cycle (day-75).

Biomarker data and correlations with response were analysed with Microsoft Excel. Comparison results from Student’s t-test with a p less than 0.05 were considered statistically significant. Kaplan-Meyer and log-rank tests were performed using GraphPad Prism 5 for Windows.

**Results:**

Regarding the response to treatment, a progression-free survival (PFS) of 9.47 months and an overall survival (OS) of 22.03 months were demonstrated. Our data are consistent with published data by other authors.

On day-15 from the beginning of the treatment an important number of patients exhibited a TSH elevation. On day-15 42.86% had a TSH over the upper normal limit and 50.0% at the end of the second cycle (day-75).

TSH increased earlier in patients that exhibited an objective response (× 3.33 times the baseline values on day-15) than patients that exhibited disease stabilisation (× 2.18) or disease progression (× 1.59). Early increases in TSH were associated with a longer PFS (11.92 vs. 8.82 months, *p* = 0.0476) and a longer OS (3.10 vs. 1.08 years, *p* = 0.0011).

**Conclusions:**

Early TSH-increase is associated with a clinical benefit. The patients that showed at least a twofold increase of their baseline TSH, responded to therapy by stabilisation or by regression of disease.

This is the only study to our knowledge which shows that early increases - 2 weeks from starting the treatment - in TSH levels have a prognostic value. Both PFS and OS of the patients who demonstrated a higher than a twofold rise were significantly longer than the PFS and the OS of the patients that presented a lower or no TSH-increase.

## Background

Malignant tumours of the kidney account for more than 3% of cancer incidents and more than 2% of cancer mortality in the European Union as estimated in 2014 [1]. In addition, during the last years, the incidence of kidney malignant tumours appears to have an increasing trend [2]. Renal cell carcinomas, representing more than 80% of all kidney tumours [3], have been treated by means of chemotherapy, interleukin 2 and pegylated interferon alfa-2b insufficiently, with response rates of  6-, 7- and 14% respectively [4–6]. New agents such as tyrosine kinase inhibitors (TKIs), have initiated a revolution in the treatment of metastatic renal cell carcinoma (mRCC) since their approval in 2006.

The results of a randomised, phase III trial comparing sunitinib with interferon (IFN)–a in treatment-naïve mRCC patients showed a statistically significant higher objective response rate (31% vs. 6%, *p* < 0.001) and a longer progression-free survival (11 vs. 5 months), with a hazard ratio of 0.42 (0.32–0.54, *p* < 0.001) [7]. However, success did not come without cost; treatment with sunitinib appeared to result in several side effects with hypothyroidism being one of the most common ones [8].

Sunitinib-induced hypothyroidism was first reported in patients with gastrointestinal stromal tumour (GIST) [9], followed by the report of Schoeffski et al referring to both patients with RCC and GIST [10] and by the report of Rini et al referring to patients with RCC [11]. The retrospective study of Rini et al demonstrates that hypothyroidism is caused by sunitinib in 85% of patients [11]. The prospective study of Wolter et al showed that only 34% of patients had no biochemical thyroid abnormality [12] while the prospective study of Baldazzi et al exhibited at least one elevated thyroid-stimulating hormone (TSH) in 59% of patients [13].

Various mechanisms such as the inhibition of thyroid peroxidase activity [14], the block of iodine uptake [due to a direct effect of sunitinib on sodium iodide symporter (NIS)], the block of TSH receptor [15], and alterations in thyroxine/triiodothyronine (T_4_/T_3_) metabolism (related to increased activity of type 3 Deiodinase) [16] have been proposed as hypothyroidism-inducing. Cases of atrophy and reduction of the thyroid gland size [17–19], degeneration of the follicular epithelial cells [17] and reduced vascularity [18] have also been related to hypothyroidism. Extra-thyroidal mechanisms may also be involved since there is a reported case of a patient treated with sunitinib who underwent thyroidectomy and developed hypothyroidism [13]. The interference of the hypothalamic-pituitary-thyroid axis is considered unrelated to hypothyroidism since no other relevant hormone abnormalities have been recorded in such cases [13, 15].

Several studies suggest that hypothyroidism is associated with cell growth inhibition and a better prognosis of different types of tumours [20–22]. An analogous correlation has been attempted between the sunitinib-induced hypothyroidism of patients with mRCC and their clinical outcome. The first report referring to this correlation was published by Wolter et al in 2002. The hypothyroid patients appeared with both longer median progression-free survival (PFS) (10.3 vs. 3.6 months) and overall survival (OS) (18.2 vs. 6.6 months) than the respective PFS and OS of patients who remained euthyroid during their treatment [12]. These results are in good agreement with the median PFS reported by Baldazzi et al. (8.55 months for hypothyroid patients vs. 7.03 months for euthyroid patients) [13]. In the study of Schmidinger et al focusing on mRCC patients under sunitinib or sorafenib, the rate of objective remission was significantly higher in the hypothyroid patients than in the euthyroid patients (28.3% vs. 3.3%) and the median duration of survival was longer (not reached vs. 13.9 months) [23]. Another study, by Riesenbeck et al*,* dealing with mRCC patients under sunitinib or sorafenib showed that a longer median PFS was related to patients who developed hypothyroidism in comparison with the euthyroid patients (16.0 vs. 6.0 months) [24]. Shinohara et al associated the high thyroid volume reduction - accompanied by hypothyroidism - in mRCC patients under sunitinib with a longer medium PFS, as opposed to the low thyroid volume reduction associated more often with euthyroidism [17]. Development of hypothyroidism was used as a prognostic parameter in the study of Sella et al as well. According to this study, hypothyroid mRCC patients under sunitinib treatment tended to have longer median PFS (12.2 vs. 9.4 months) and longer OS (22.4 vs. 13.9 months) than euthyroid patients. Both groups appeared to have similar clinical benefit [25]. Nevertheless, the prospective study of Sabatier et al reported no association between mean PFS and hypothyroidism [26].

We evaluated the impact of hypothyroidism and early changes in TSH levels in the treatment outcome of 70 mRCC patients treated with sunitinib.

## Methods

### Eligibility criteria

Seventy patients with histologically confirmed metastatic clear-cell RCC were enrolled in our retrospective study. They were consecutive patients as appeared on the database of our Renal Cancer Clinics. Based upon the Eastern Cooperative Oncology Group (ECOG) criteria, all patients had a performance status of ≤2 and they were aged between 18 and 80 years old. For these patients, sunitinib was used as first line therapy. All patients had adequate bone marrow (Haemoglobin ≥10.0 g/dL, White Blood Count ≥3000 × 10^9^/L, Neutrophils ≥1000 × 10^9^/L and Platelets ≥100,000/mcL), renal (Creatinine ≤2 mg/dL), hepatic (transaminases ≤3 times the upper limit of normal values, total bilirubin ≤2 times the maximum limit of normal limit) and cardiac (Left Ventricular Ejection Fraction at least 50%) function. Patients receiving any medication known to intervene with thyroid function or who had received external neck irradiation or radioiodine therapy were excluded from the analysis.

### Drug administration

Sunitinib was administered in accordance with the conventional 6-week schedule which consisted of 4 weeks of daily administration of 50 mg, followed by a 2-week off-treatment interval. Dose reduction (37.5 mg with the same 4-weeks on and 2-weeks off schedule) was allowed according to the side-effect profile. The patients have been treated with sunitinib from the day of diagnosis until the date of radiologically confirmed relapse or the date they dropped out due to side effects.

### Examinations on treatment

Physical examination, performance status, blood cell counts and serum chemistry were assessed at baseline, on day − 15, − 30, − 45, − 60, − 75, − 90 as well as at the beginning and end of the following treatment cycles thereafter. The National Cancer Institute Common Terminology Criteria for Adverse Events version 3.0 were utilised to grade both adverse effects and abnormal laboratory values. Tumour evaluation was performed by means of computed tomography or magnetic resonance imaging as well as bone scintigraphy where needed before the start of sunitinib therapy and at the end of every two cycles. Clinical outcome was assessed by CT and MRI scans, and reported using the RECIST criteria [27].

### Evaluation of thyroid function

The thyroid function of all patients was appraised at baseline and on days − 1 and − 28 of each treatment cycle by means of TSH and free T_4_. Notably at baseline, on day-15 of the first cycle and on day-28 of the second cycle a number of parameters were evaluated; TSH (reference range 0.30–4.0 mU/L), free T_4_ (reference range 7.8–19.4 pg/mL), antibodies against thyroid peroxidase (TPOAb; reference range ≤ 50 U/mL), antibodies against thyroglobulin (TgAb; ≤70 U/mL) and antibodies against the TSH receptor (TR-Ab; reference classification as "negative" when < 9 U/L, "questionable positive" when 9–14 U/L, "positive" when > 14 U/L). All serum samples were assayed by immunoradiometric (IRMA) methods and tests were performed in duplicate. Specifically, for TSH and free T_4_, kits by DiaSorin, Saluggia, Italy were used, while for TPOAb, TgAb and TR-Ab, kits by ZenTech, Angleur, Belgium were used.

According to the guidelines of the American Association of Clinical Endocrinologists and the American Thyroid Association, subclinical hypothyroidism is connected with a serum TSH above the upper reference limit combined with a free T_4_ into the normal reference range, while overt hypothyroidism is characterised by an elevated TSH in combination with a subnormal free T_4._

Thyroid replacement with levothyroxine was initiated at the discretion of the physician, based on both serum TSH measurements and any clinically significant symptoms related to hypothyroidism such as fatigue, cold intolerance, weight gain and constipation. The aim of the thyroid replacement was a TSH serum concentration between 0.50 and 2.50 mU/L.

### Bioanalytics

The blood sample collection was followed by their transport on ice and centrifugation in order to separate serum. The storage of the aliquots was carried out at -80^o^ C and they were thawed only once.

### Statistical analysis

Biomarker data and correlations with response were analysed with Microsoft Excel. Comparison results from Student’s t-test with a *p* value less than 0.05 were considered as statistically significant. Kaplan-Meyer and log-rank tests were performed using GraphPad Prism 5 for Windows.

## Results

### Patient characteristics

We included 70 patients (54 male and 16 female) diagnosed with metastatic clear-cell RCC. The median age of the patients was 62 years (age range between 25 and 79 years old). Most patients had an intermediate prognosis according to MSKCC criteria (41 patients, 58%), with the rest having poor prognosis (25 patients, 36%) and a few favourable (4 patients, 6%) (Table 1).

**Table 1:** Patient characteristics

Patient characteristics	Absolute number	%
Number		
Total included	70	100
Evaluable for response	62	88
Sex		
Male	54	77
Female	16	23
Age		
Median	62	
Range	25 – 79	
Performance Status		
0	30	43
1	28	40
2	12	17
MSKCC risk classification		
Favourable	4	6
Intermediate	41	58
Poor	25	36

Characteristics of all patients enrolled in the study

### Response to treatment

From the 70 patients who participated in the study, 62 were evaluable for response; 50 patients had a clinical benefit (CB) [2 patients had complete response, 24 patients had a partial response (PR) and 24 patients had stable disease (SD)] and 12 patients exhibited disease progression (DP) during or after 2 cycles of treatment and discontinued.

**Fig. 1:**
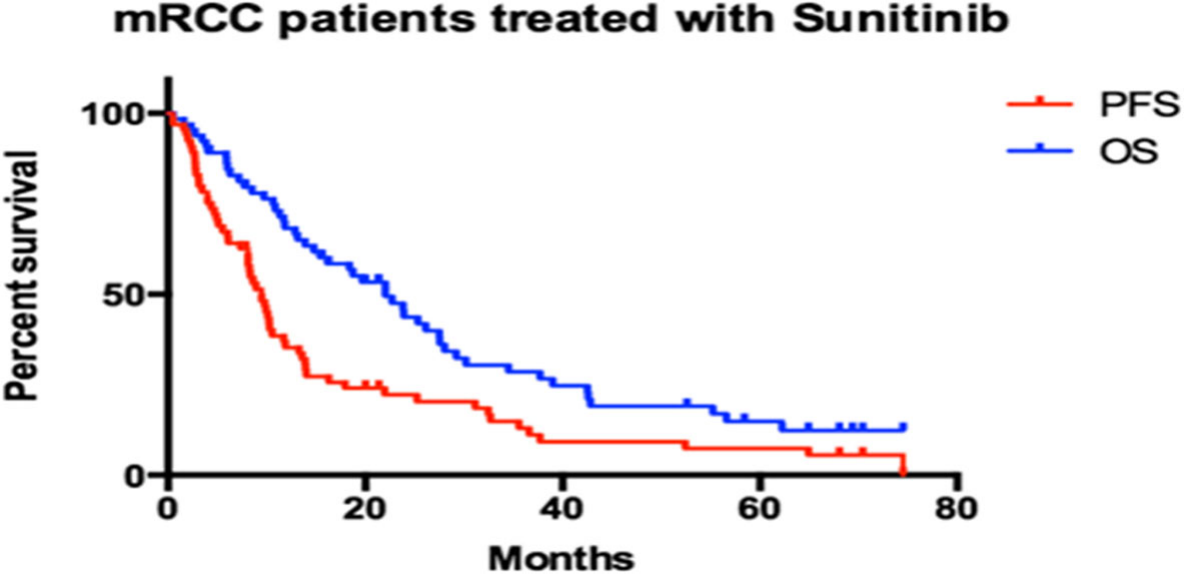
Response to treatment. In our cohort the median PFS was 9.47 months and the median OS was 22.03 months. Our data are consistent with published data by other authors. The median follow-up of our patients was 19.2 months

Median progression-free survival (PFS) was 9.47 months whilst median overall survival was 22.03 months (Fig. 1). Median follow-up was 19.2 months.

Following relapse, m-Tor inhibitors, namely temsirolimus and everolimus, have been commenced as salvage therapy.

### Thyroid function during treatment

All patients had an assessment of their thyroid values at baseline. Three patients were diagnosed with subclinical hypothyroidism.

All patients received sunitinib treatment and were reassessed for thyroid function 15 days later.

Hypothyroidism arose earlier than expected, since 30 patients (42.86%) had a TSH over the upper normal limit, only 15 days after the beginning of sunitinib therapy. At the end of the second cycle (day-75) 35 patients (50.0%) were reported to have a TSH-increase above the higher normal value. The median TSH concentration at baseline was 1.96 mU/L and the average TSH concentration was 2.79 mU/L. On day-15 the median TSH level was 3.88 mU/L and the average TSH level was 5.50 mU/L, while on day-75 it was 5.19 mU/L and 10.04 mU/L respectively. All free T_4_ values were determined in the normal range, except one case of a patient that had a TSH concentration of 60.64 mU/L with a free T_4_ value of 7.12 pg/mL (day-75). From the patients who developed hypothyroidism during treatment, only 2 were found with elevated titres of TPOAb, 2 patients with increased TgAb and 1 patient with both antibodies over the normal limit. Moreover, 2 patients developed higher than normal TR-Ab, 1 of whom was diagnosed with hypothyroidism. Depending on the TSH levels in combination with a hypothyroid-related clinical status, levothyroxine was prescribed in order to achieve a TSH value between 0.50 and 2.50 mU/L.

### Thyroid function and response to treatment

**Fig. 2:**
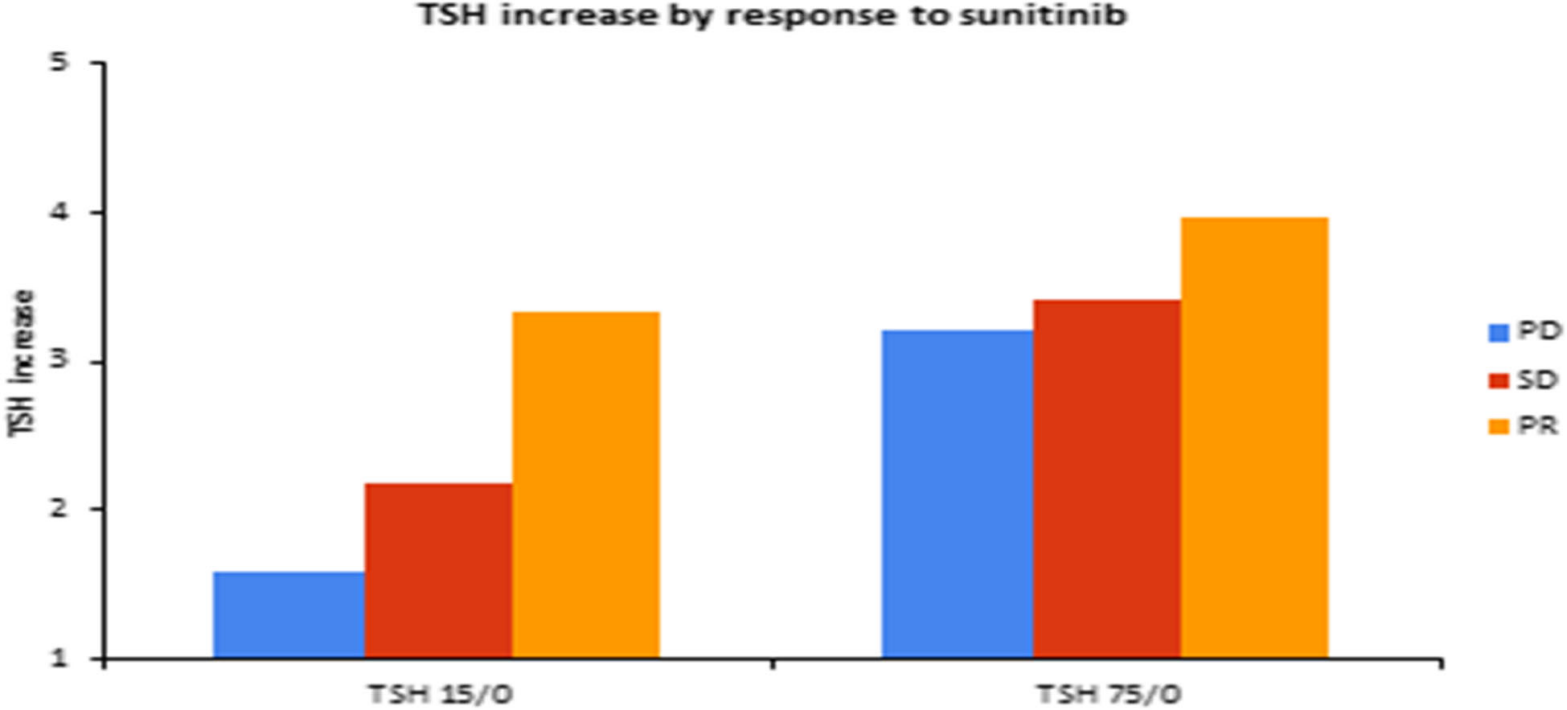
Association of TSH increase to treatment response. On day-15 there was a mean increase × 2.52 the baseline value (paired t-test *p* < 0.00001) and on day-75 (by end of cycle 2) × 3.68 the baseline value (paired t-test *p* < 0.00001). TSH increased earlier (day-15) in patients that exhibited a response: × 1.59 times in PD patients, × 2.18 times in SD patients and × 3.33 times in PR patients on day-15. These differences were less prominent on day-75 (× 3.29, × 3.41, and × 3.96 respectively). The data suggest that early increase in TSH levels may predict clinical benefit from sunitinib in mRCC patients

On day-15 there were only 8 patients that experienced a decrease in TSH while the majority had increased TSH levels. Mean TSH increase was 2.52 times baseline values (paired t-test *p* < 0.00001) after 15 days of treatment and 3.68 times baseline values after 75 days of treatment (by end of cycle 2, paired t-test *p* < 0.00001). TSH increased at an earlier time point (Fig. 2) in patients that exhibited an objective response (× 3.33 times the baseline values on day-15) than patients that exhibited disease stabilisation (× 2.18) or disease progression (× 1.59). These differences were less prominent on day-75 (3.19, 3.41 and 3.96 respectively]. Overall, these data suggest that early increase in TSH levels may predict clinical benefit from sunitinib in metastatic RCC patients.

### Impact of hypothyroidism on survival

**Fig. 3:**
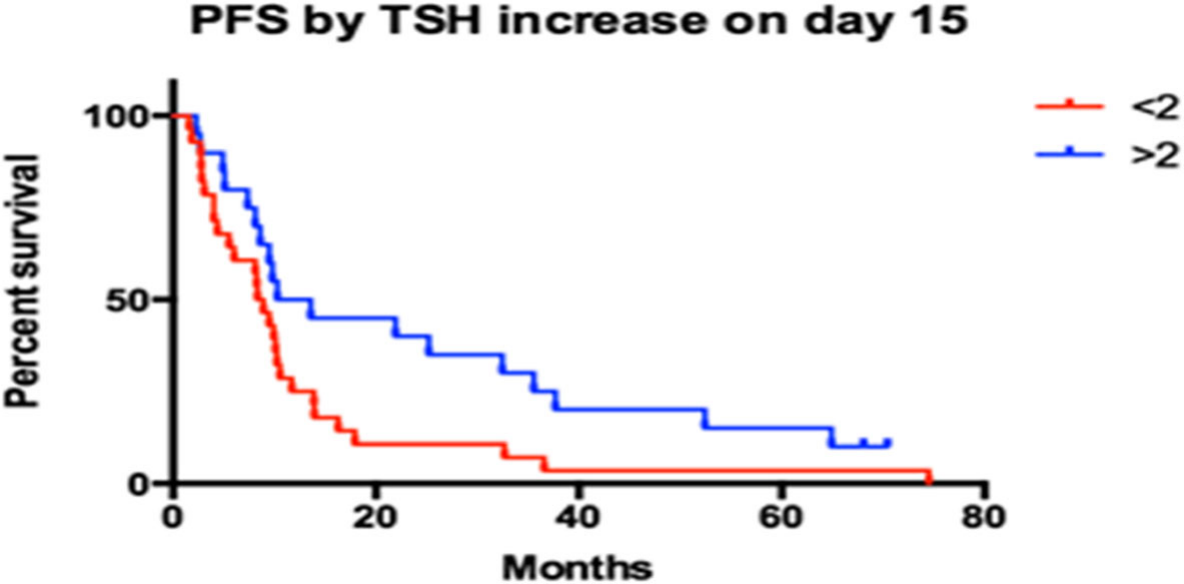
Early increase in TSH predicted longer PFS. Patients with a TSH increase > 2 x baseline value on day-15 exhibited a longer PFS than those with a TSH increase less than twice their baseline value (11.92 vs 8.63 months, HR 1.76, 95% CI: 1.033–3.288, *p* = 0.0476) (Log-Rank test)

**Fig. 4:**
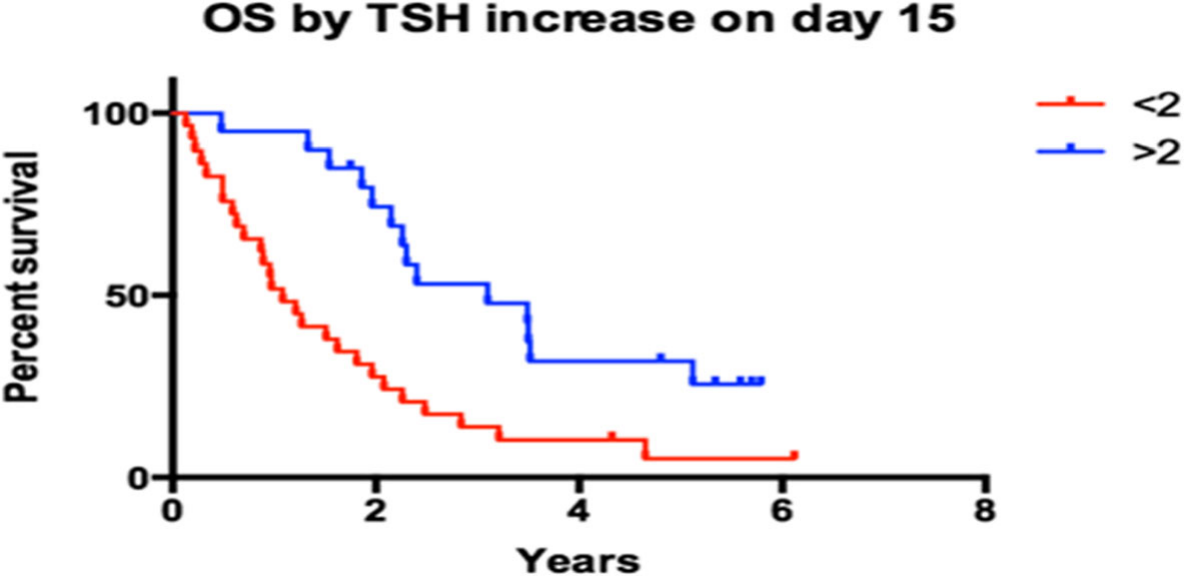
Early increase in TSH predicted longer OS. Patients with a TSH increase more than twice their baseline value on day-15 exhibited a longer OS than those with a TSH increase less than twice their baseline value (3.1 vs 1.08 years, HR 1.73, 95% CI: 1.556–5.394, *p* = 0.0011) (Log-Rank test). The result is statistically significant

We analysed the association between early increase in TSH levels and progression-free or overall survival. There was a statistically significant association between TSH increase and PFS on first line sunitinib as seen in Fig. 3. Median PFS was 11.92 months for patients that had more than double the baseline TSH levels and 8.63 months for patients that had a day-15/baseline TSH ratio smaller than 2. The difference was statistically significant with a *p* value of 0.0476 (log-rank test) and a hazard ratio of 1.76 (95% CI: 1.033 to 3.288). However, early TSH increase had a more prominent impact on overall survival (Fig. 4). Median OS was 3.10 years in patients that had a day-15/baseline TSH ratio of > 2 and 1.08 years for those whose ratio was < 2. The hazard ratio was 1.73 (95% CI: 1.556 to 5.394) and the result was statistically significant with a *p* value of 0.0011 (log-rank test). There were no prognostic factors that exhibited impact on response other than the established ones such as the MSKCC criteria. However, the impact of early increase of TSH levels had a prognostic effect throughout all MSKCC categories.

## Discussion

The study addresses the following three points: the response of the mRCC patients to the treatment with sunitinib, the manifestation of sunitinib-induced TSH increase and, by extension, the appearance of hypothyroidism and finally the influence of the latter on the patients’ survival.

Regarding the response to sunitinib treatment, the results of this study demonstrate a progression-free survival of 9.47 months and an overall survival of 22.03 months, in concert with our previously published results [28].

Hypothyroidism has been reported as a typical and usual side effect of the sunitinib treatment by many writers in both retrospective and prospective studies. In our study, we observed that 42.86% of the patients were already hypothyroid only 2 weeks after starting sunitinib. Hypothyroid status becomes more common as sunitinib treatment is continued and 50% of the patients were hypothyroid at the end of the second therapy cycle. The fact that roughly all members of the cohort did not have abnormal thyroid antibody levels, coincides with the conclusion of many studies that autosomal mechanisms do not play a critical role in the emergence of hypothyroidism [11, 12, 16, 29]. The physiological free T_4_ titres in all patients – with the exception of one case – verify the occurrence of subclinical hypothyroidism and the need of thyroid supplementation with levothyroxine depending mainly on the clinical picture.

A considerable number of studies have correlated the presence of hypothyroidism with a longer PFS and OS, as previously reported. In our study, we noticed that early TSH-increase is associated with a clinical benefit. At the end of the second week from the beginning of the treatment, an important number of patients exhibited an elevation of TSH values. The patients that showed at least a twofold increase of their baseline TSH titres responded to therapy by stabilisation or by regression of disease. On the other hand, patients with a less remarkable or non-elevated serum TSH had a higher chance to experience disease progression after two cycles of treatment.

 A highly significant observation in our study is  that the magnitude of TSH increase in the very early stages of treatment with sunitinib (day-15) appears to have a prognostic value. The PFS of the patients who demonstrated a higher than a twofold rise was significantly longer than the PFS of the patients that presented a smaller or no TSH-increase and this was reflected to overall survival. As the increase in OS is much more prominent than the increase in PFS we have to exclude the hypothesis of a pharmacodynamic or pharmacokinetic effect of sunitinib treatment and rather generate a hypothesis of a “class effect” in thyroid function (as > 30% of our patients received another RTKI in the 2nd or 3rd line) or a mere effect of hypothyroidism on mRCC (as suggested by Schmidinger et al).

## Conclusions

Our study reports a PFS and an OS for mRCC patients treated with sunitinib which are in concordance with previous studies. Hypothyroidism is a common side effect associated with sunitinib and this study demonstrated a particularly early onset. Hypothyroidism occurred only 2 weeks after commencing sunitinib while its occurrence in the population studied increased further by the end of the second therapy cycle. This early TSH-increase is related to a clinical benefit; specifically, those with at least a twofold increase of their baseline TSH titres responded to therapy by stabilisation or by regression of disease in comparison to those with a smaller or no increase of their baseline TSH who demonstrated a higher chance of disease progression. Finally, both the PFS and the OS of patients who presented a higher than a twofold rise of their baseline TSH on day-15 were significantly longer than the PFS and the OS of patients that exhibited a smaller or no increase of their baseline TSH, with the OS prolongation being more evident.

## Abbreviations

CB: Clinical benefit
DP: Disease progression
GIST: Gastrointestinal stromal tumour
IFN: Interferon
IRMA: Immunoradiometric assay
mRCC: Metastatic renal cell carcinoma
NIS: Sodium iodide symporter
OS: Overall survival
PFS: Progression-free survival
PR: Partial response
SD: Stable disease
T_3_: Triiodothyronine
T_4_: Thyroxine
TgAb: Antibodies against Thyroglobulin
TKIs: Tyrosine kinase inhibitors
TPOAb: Antibodies against thyroid peroxidase
TR-Ab: Antibodies against TSH receptor
TSH: Thyroid - stimulating hormone
